# The biomechanical differences of rotational acetabular osteotomy, Chiari osteotomy and shelf procedure in developmental dysplasia of hip

**DOI:** 10.1186/1471-2474-15-47

**Published:** 2014-02-21

**Authors:** Ming Fu, Shanshan Xiang, Zhiqi Zhang, Guangxin Huang, Jin Liu, Xin Duan, Zibo Yang, Peihui Wu, Weiming Liao

**Affiliations:** 1 Department of Joint Surgery, First Affiliated Hospital, Sun Yat-sen University, Guangzhou 510080, China; 2 Department of Orthopaedic Surgery, the Affiliated Jiangmen Hospital of Sun Yat-sen University, Jiangmen 529070, China

**Keywords:** Rotational acetabular osteotomy, Chiari osteotomy, Shelf procedure, Hip stress

## Abstract

**Background:**

Rotational acetabular osteotomy (RAO), Chiari osteotomy and shelf procedure are important treatments to delay the progression of osteoarthritis in developmental dysplasia of hip (DDH) patients, but their biomechanical differences are still unknown. This study was to evaluate the different biomechanical changes of hip joint after these three surgeries.

**Methods:**

Sixteen DDH models of 8 human cadaver specimens were reconstructed, and treated by different surgeries, and then strain around femoral head was evaluated by strain gauges.

**Results:**

Hip strain value of DDH model was decreased after treated by shelf procedure (Pleft = 0.016 and Pright = 0.021) and rotational acetabular osteotomy (P = 0.004), but not in Chiari osteotomy (P = 0.856). Moreover, the improved ratio of RAO treatment was better than shelf procedure (P = 0.015) and Chiari osteotomy (P = 0.0007), and the descendent range of shelf procedure was greater than Chiari osteotomy (P = 0.018).

**Conclusions:**

From biomechanics points, RAO was more effective in relieving hip joint stress compared with shelf procedure and Chiari osteotomy.

## Background

Developmental Dysplasia of Hip (DDH) is a common congenital deformity of hip. If appropriate measures are not taken, DDH often develops into secondary hip osteoarthritis (OA) due to the abnormal stress of hip [1-5]. Although total hip arthroplasty (THA) is an effective treatment for late stage OA, its application for young DDH patients is still controversial in consideration of the survival rate of prosthesis [6,7]. It is widely accepted that joint preserving operations such as periacetabular osteotomy should be selected whenever possible [8-12]. Rotational acetabular osteotomy (RAO), Chiari osteotomy and shelf procedure are three different periacetabular osteotomies, which are often used for adolescent and adult DDH. All of them could re-adjust the positional relationship of the acetabulum and femoral head to increase the acetabular coverage of the femoral head, restore normal anatomical structures, and then improve the biomechanical properties of hip. Previous studies often focused on the clinical outcomes of these three procedures [13-19], while relatively little knowledge is available related to the postoperative biomechanical changes of hip. Iliescu N et al. reported that the principal stress on the surface of the acetabulum was decreased by 17.4% after Chiari osteotomy in a photoelastic investigation, although it was still greater than a normal hip [20]. And it was shown on a computer model that increasing the height of Chiari osteotomy from acetabular rim decreased the load-bearing contact area as well as increased the contact pressure of hip [21]. However, the different biomechanical changes among these three operations have not been reported before.

In this study, RAO, Chiari osteotomy and shelf procedure were performed on sixteen DDH models which were established on eight adult cadaver pelvis specimens. Hip strain was measured before and after these three osteotomies in an attempt to investigate the different biomechanical changes of hip after operations and compare their efficiency in reducing the abnormal stress of hip in DDH.

## Methods

### Subjects

This study was approved by the Medical Ethics Committee of Sun Yat-sen University. Twelve female cadavers who had died at 20 to 40 years old were offered by the Department of Anatomy of Sun Yat-sen University. Anteroposterior diameters of the pelvic inlet (the distance from midpoint of superior margin of pubic symphysis to midpoint of superior margin of promontory) and transverse diameters of the pelvic inlet (the maximum distance between left and right iliac crest) were measured. Eight pelvic specimens were selected because their anteroposterior diameters were 10.5 cm ~ 11.6 cm and the transverse diameters were 12.6 cm ~ 13.8 cm which showed these eight specimens were within a 5% of variation in pelvic bone size. The fifth lumbar vertebrae, the hip joint capsule and the proximal and middle portion of femur in every specimen were kept. Abnormalities, tumors and damages of pelvis were excluded by general observation and X-ray examination.

### Methods

#### Measurement of normal specimens

**Figure 1:**
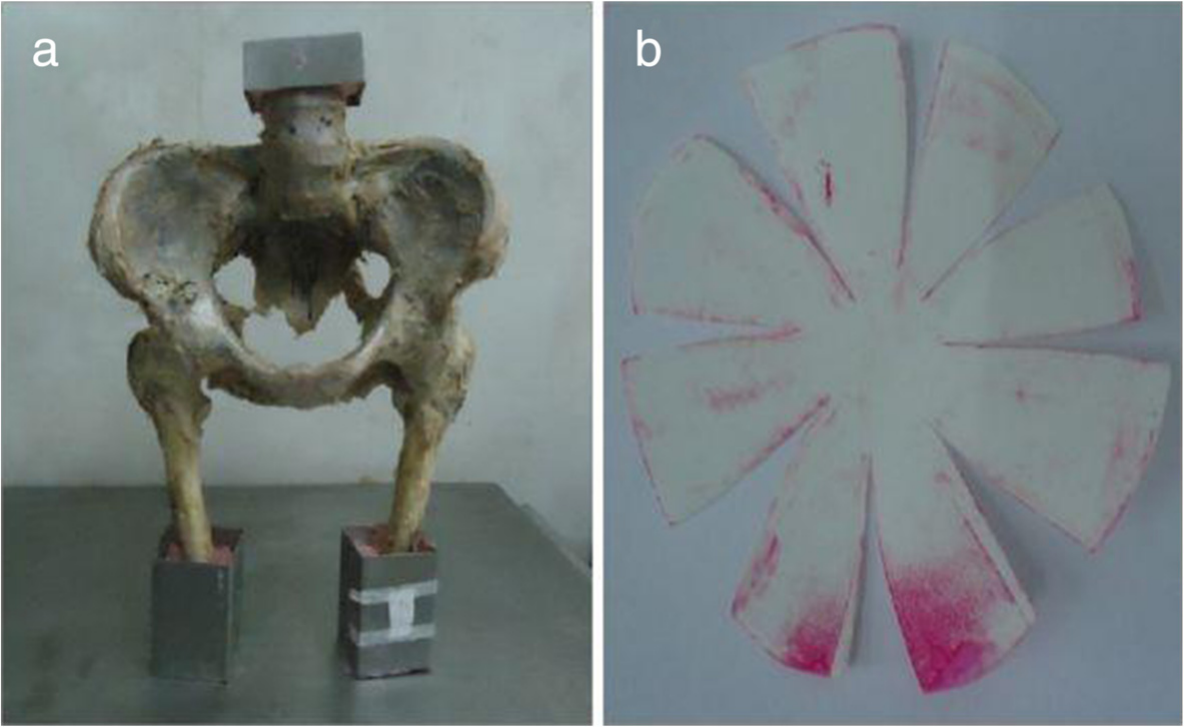
**Fixation of pelvic specimen and color change of the Fuji film. a.** the longitudinal axis of femoral diaphysis and median line of pelvis was in 15° adduction angle. **b.** the area of Fuji film where color change was most obvious.

After rejecting adherent soft tissue all pelvic specimens were entrapped within the homemade fixture with denture acrylic (Shanghai medical apparatus and instruments Ltd, China). The anterior superior iliac spine and the pubic symphysis plane of each specimen were kept in the same coronary and femurs were held in 15° adduction angle in order to simulate human standing posture [22] (Figure 1a). Hip joint capsule was incised from lateral, and femoral head was cleaned up after it was dislocated so that the pressure sensitive paper (prescale low pressure (LW): 2.5-10Mpa, Fujifilm, Japan), which was cut according to the shape of each femoral head, could completely cover it [23]. Following that, femoral head was reduced and the specimen along with the fixture was installed on the material testing machine (SANS CMT6104, MTS Company, USA). Then, a hydraulic load at a speed of 5 mm/min was applied on the preload of 200 N to detect the maximum stress region of femoral head [24]. The region was positioned according to the corresponding area where the discoloration was most obvious on the pressure sensitive paper which was removed from femoral head gently (Figure 1b). Then as we reported previously [24], the center of the maximum stress region of femoral head was marked for a strain gauge (KFG-5-120-C1-11L3M2R, sensitivity coefficient: 2.08 ± 1.0%, KYOWA Company, Japan) in biaxial. The shape of the strain gauge, 15 mm × 5 mm in size and 33 μm ~ 38 μm in thickness, was adjusted with dimensions base on the curve of femoral head. It was pasted onto articular cartilage at the marked central region of the maximum stress area using quick-drying adhesive (cyanoacrylate adhesive CC-33A, KYOWA Company, Japan) without any bubble. And it could cover 34% of the half sphere in the mediolateral direction of femoral head. Then an accessory film of polyethylene resin from KYOWA was used to cover the strain gauge. A latex cover was placed over the strain gauge, and waited for about 60 minutes until the adhesive was completely harden. Afterward the specimen was fixed in the material testing machine again and the hip strain value was measured with KYOWA PCD-2300A strain data analysis system (KYOWA Company, Japan) when a hydraulic load was applied at a speed of 5 mm/min until 600 N [25]. Every hip was measured three times.

#### Establishment and measurement of DDH model

**Figure 2:**
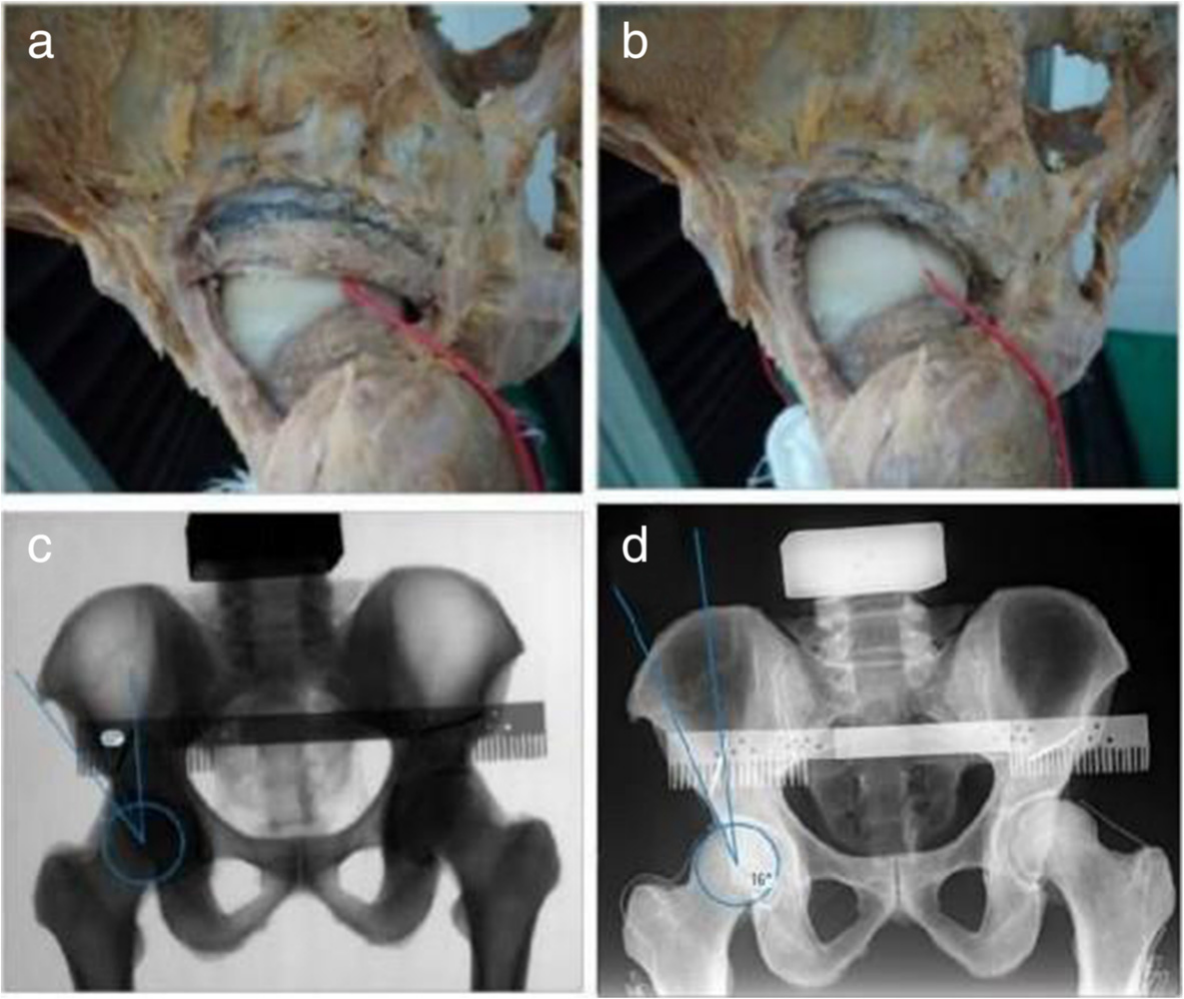
**The model of DDH. a** and **b** were the appearance of specimen before and after osteotomy; **c** and **d** were the X-ray pictures before and after osteotomy, and the CE angle was 32°before osteotomy, and it turned to 16°after osteotomy.

X-ray of pelvis was taken for each specimen and Wiberg central-edge angle (CE angle) was measured on the X-ray film. Firstly, a homemade metallic scale with 2 mm accuracy of measurement was fixed just above the acetabulum of the specimen using two Kirschner wires. X-ray was took repeatedly to ensure the lower edge of the scale was parallel to the connecting line between bilateral acetabular upper edges, so that it could be used as the reference when making a dysplastic hip (Figure 2c). Then we drew a predicted 1.0 cm bone-cut line on the posterior and superior border of acetabulum (Figure 2a). After resecting the bone with a chisel [26] (Figure 2b), the pelvis X-ray film was retaken to affirm that the CE angle of the build hip was 10 to 20 degrees (Figure 2d). So our model was consistent with DDH in morphology and imaging examination. Then the maximum stress region of femoral head of the DDH model was located using the method mentioned above. Similarly, the DDH model was fastened on the material testing machine and hip strain value was measured three times in the aforementioned way.

#### Performance of three osteotomies and measurement

**Figure 3:**
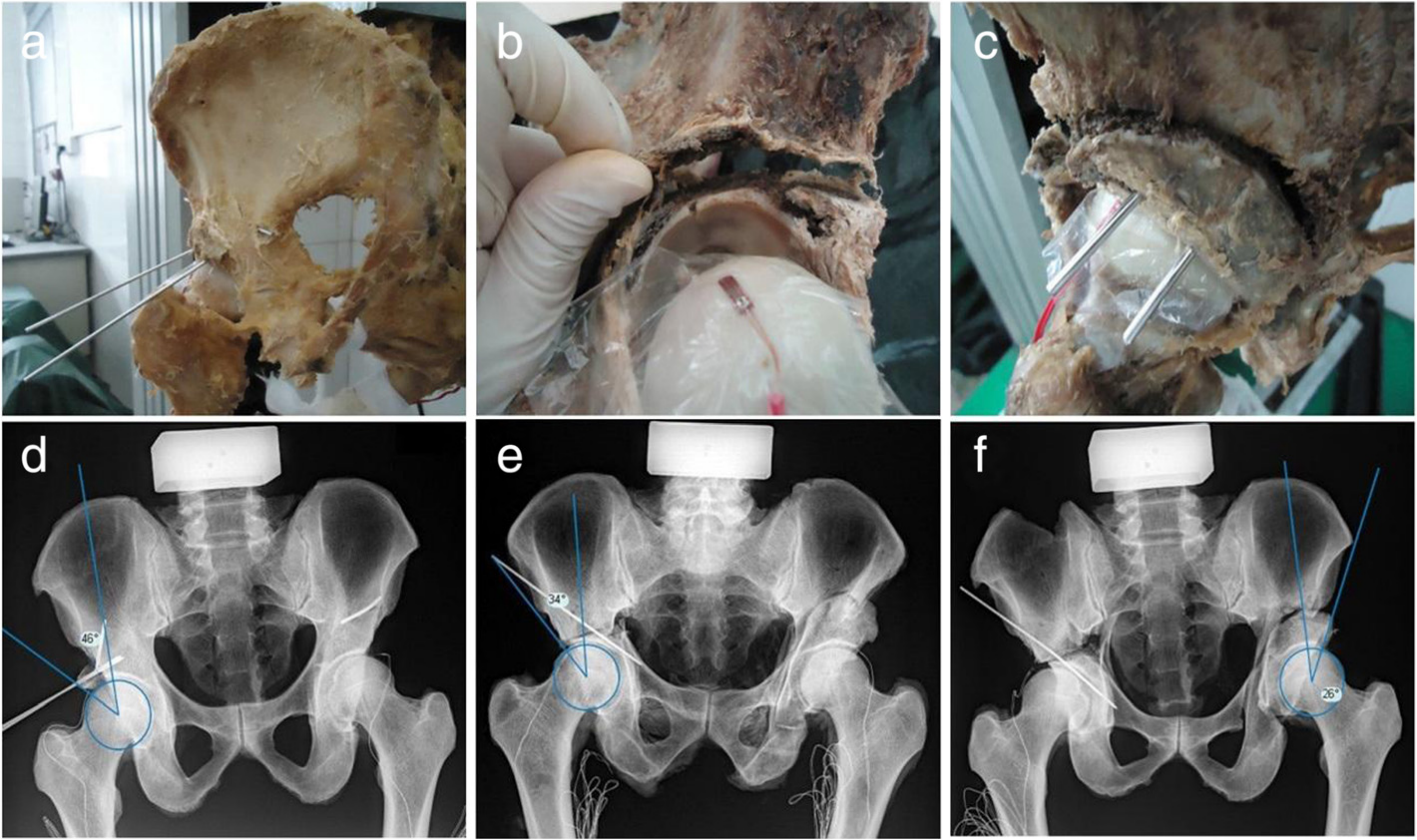
**The three different treatment options for DDH. a-c** were the appearance of specimen after shelf procedure **(a)**, Chiari osteotomy **(b)** and RAO **(c)**; d-f were the X-ray pictures after shelf procedure **(d)**, Chiari osteotomy **(e)** and RAO **(f)**, and CE angles after every osteotomy were all greater than 20 degrees.

After establishment and measurement of DDH model, shelf procedure, Chiari osteotomy and RAO were performed on the specimens by the same surgeon (S.X.). The shelf procedure was carried out on both hip joints of each model and the bone fragments of width 1.0 cm were used (Figure 3a). After removing the bone fragments of shelf procedure, Chiari osteotomy was performed on the right hip of each specimen and RAO was performed on the left hip (Figure 3b and c). For Chiari osteotomy, because the maximal movement distance was 1.0 cm due to its own limitation of antiseptic cadaver specimen, the bone fragment was tried to be moved inward by 1.0 cm. At the same time, the bone fragment was rotated outward by 1.0 cm in RAO. X-ray of pelvis was taken and CE angles were confirmed in the normal range after every procedure (Figure 3e-f). The pressure sensitive paper was used to detect the maximum stress region of femoral head after each osteotomy and hip strain value of the detected region was obtained as described above.

### Statistical analysis

All data were presented as mean and standard deviation. We assumed that hip strain value of the DDH model before osteotomy was α and the value after osteotomy was β, and then the improved ratio of osteotomy of hip strain of DDH model was equal to (α-β)/α. The differences of hip strain values before and after osteotomy within the same group were compared with paired-sample *T* test. One-way analysis of variance was applied in comparison among these three groups and SNK test was adopted in comparison between any two groups. All statistical analyses were performed using SPSS version 13.0 and *P* values <0.05 were considered statistically significant.

## Results

The maximum stress region of femoral head was consistent in all normal pelvis specimens, all of which were located on the site corresponding to the posterior border of acetabulum. Comparing with normal specimens, the maximum stress region of DDH model was shifted outward the site where the femoral head contacted with posterior border of acetabulum because CE angle became smaller.

The strain values of left and right hip in normal specimens were 646.88 (SD 789.98) and 955.94 (SD 837.42) respectively, while they were obviously increased about two times in DDH models to 1439.03 (SD 625.23) and 1558.75 (SD 1009.46) (Tables 1 and 2). According to the morphology, imaging examination and these biomechanical results, the DDH model was considered to be successful.

**Table 1:** The relative strain values of normal specimens before and after establishment of DDH (left) (10–6)

**NO.**	**A**	**B**	**C**	**D**	**E**	**F**	**G**	**H**	**Average**
Before	720.00	370.00	1912.50	-795.00	1027.50	1125.00	650.00	165.00	646.88
After	1126.25	940.00	2386.00	932.50	1245.00	2420.00	1505.00	957.50	1439.03
After/Before	1.56	2.54	1.25	1.17	1.21	2.15	2.32	5.80	2.25

**Table 2:** The relative strain values of normal specimens before and after establishment of DDH (right) (10–6)

**NO.**	**A**	**B**	**C**	**D**	**E**	**F**	**G**	**H**	**Average**
Before	957.50	225.00	842.50	952.50	2900.00	700.00	250.00	820.00	955.94
After	1005.00	540.00	2235.00	1175.00	3395.00	905.00	735.00	2430.00	1552.50
After/Before	1.05	2.40	2.65	1.23	1.17	1.29	2.94	2.96	1.96

**Figure 4:**
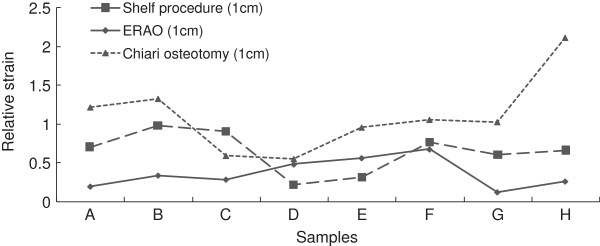
Strain trend of femoral heads in different surgical osteotomies for DDH models.

After shelf procedure, the strain value of left hip was decreased to 1083.13 (SD 784.51) and that of right hip was dropped to 816.88 (SD 671.03), both of which had significant differences before and after the procedure (P1eft = 0.016 and Pright = 0.021). Likewise, the strain value of left hip was significantly declined to 574.94 (SD 430.88) after RAO (P = 0.004). However, the average strain value of right hip was raised to 1614.81 (SD 932.67) after the Chiari osteotomy because five of these hips’ strain values were increased by 268.00 ~ 1149.50, averaged 622.70. Nevertheless, no significant difference of hip strain value was found before and after Chiari osteotomy (P = 0.856). Moreover, the improved ratio of RAO treatment was better than shelf procedure significantly (P = 0.015) and Chiari osteotomy (P = 0.0007), and the descendent range of shelf procedure was greater than Chiari osteotomy significantly (P = 0.018) (Figure 4).

## Discussion

DDH patients with acetabular and femoral head developmental defect often have an abnormal shape and/or altered position of acetabulum, which leads to insufficient covering of femoral head or mismatch of femoral head and acetabulum. Consequently, the anatomical basis that maintains hip biomechanical stability changes thereby makes the femoral head prone to lateral displacement and secondary subluxation. These changes in anatomical structure result in reduction of loading area of hip and obvious increase of the tension, pressure and shear stress of articular surface. Comparing with normal hip, hip of DDH has a 26% reduction of the contact area and a 23% increment of pressure [27]. Osteoarthritis occurs when the pressure exceeds the allowable range of osteoblasts and chondrocytes. Rotational acetabular osteotomy, Chiari osteotomy and shelf procedure are three effective treatment options for DDH, which could delay the onset of secondary osteoarthritis. Here, we identified the trend of biomechanical change on these three acetabular osteotomies, through establishing sixteen DDH models of human cadaver specimens.

The shelf procedure could alleviate the hip pressure and reduce the tension of the capsule without causing hip stiffness postoperative. And this operation is relatively simple and safe without disruption of the pelvic ring structure. Hence, it can be used for young patients with subluxation of hip, providing good stability and mobility, postponing subsequent arthroplasty. Moreover, because it merely affects the hip structure, it would not increase the difficulty of the arthroplasty. However, the procedure has the risk of bone resorption and collapse. After a follow-up of 140 patients 157 hips for 5 years, Staheli found that 83.3% of the patients had a satisfactory hip function recovery after the shelf procedure. In our experiment [28], we found that the shelf procedure could obviously reduce hip stress of DDH.

In a follow-up study of 96 DDH patients 100 hips after Chiari osteotomy, Matsuno found that 84% patients’ clinical symptoms improved postoperatively [29]. Chiari osteotomy is intended to move the hip inward from osteotomy line in order to increase the bony coverage of femoral head. However, the increased coverage does not match the shape of femoral head. Our results showed no significant change in hip stress before and after Chiari osteotomy. Even five of these specimens had an increased hip stress postoperatively. One possible reason for this outcome is that when the hip was moved inward, femoral head became closer to the center of gravity line which changed the conducting direction of weight-bearing force and raised the pressure of hip. Whereas our experiment was a biomechanical study within a short period after osteotomy, it could not reveal the long-term effect of Chiari osteotomy. In clinic, the capsule could turn to metaplastic fibrocartilage and then form a new acetabulum over long term, which could expand the contact area of femoral head and acetabulum and distribution of hip stress. Consequently, function of hip was improved and the occurrence of osteoarthritis was delayed. It had been reported that as a result of the establishment of a larger contact zone, the contact pressure distribution of the hip was close to normal two years after Chiari osteotomy in a photoelastic study [20]. Even so, whether biomechanical property of hip could be improved in vivo when the metaplastic fibrocartilage becomes a part of acetabulum still remains to be further studied.

After RAO, the position relation between acetabulum and femoral head is readjusted to increase acetabular coverage of femoral head and reduce acetabular shear force that lead to restoration of normal hip joint anatomy and biomechanical properties. Previous studies have reported that RAO could obviously improve clinical symptoms and hip function of DDH patients and achieve satisfactory clinical outcomes [30]. Our data suggested that RAO could greatly reduce hip stress of the DDH model due to congruence between acetabulum and femoral head postoperatively, which was better than shelf procedure and Chiari osteotomy. From another aspect, unlike the long term articular cartilage after Chiari osteotomy which consists of fibrocartilage transformed from joint capsule, the articular cartilage after RAO is the own hyaline cartilage of the hip with better tissue compatibility so that RAO could greatly delay the progression of osteoarthritis. It was reported that about 80% patients who underwent this operation avoided total hip replacement in the future [31]. While similar studies of Chiari osteotomy and shelf procedure were so far lacking. Therefore, we believe that for young DDH patients who need surgical interventions but not to the point at which the arthroplasty is required, RAO may be a better choice with greater advantages.

However, there are some limitations in this research. First of all, this is a measurement study base on cadaver specimens, and thus the data had certain difference from real human. Next, the biomechanical characteristic of human body is so complicated that it cannot be real simulated completely in vitro. And strain measurement at only one time-point is insufficient because biological effects are always different at various points in time. In addition, there was only one strain point in this research, but this point was located in the maximum stress region of femoral head, so it was able to reflect the force condition of hip to some extent. Certainly, we will also design more strain points to better reflect the force condition in further study. As we focused on the hip stress when standing, the changes of hip stress in different directions after three kinds of surgical methods require further exploration combined with finite element analysis and detection of muscle force. Furthermore, the ideal strain gage should change resistance only due to the deformations of the surface to which the sensor is attached. However, in real applications, temperature, material properties, the adhesive that bonds the gage to the surface, and the stability of the metal all will affect the detected resistance. Therefore, all of these limitations should be corrected in our future research.

## Conclusions

In summary, from our findings, RAO and shelf procedure, especially the former are effective treatments for DDH in the biomechanics.

## Competing interests

The authors declare that they have no competing interests.

## Authors’ contributions

M.F. and S.X. carried out the majority of the experiments, analyzed data and prepared the manuscript; Z.Z. and G.H. assisted with the experiments and the analysis of the data; J.L., X.D., Z.Y., P.W. and W.L. provided suggestions for the study and critically reviewed the manuscript; M.F. and Z.Z. supervised the project and wrote most of the manuscript. All authors read and approved the final manuscript.

## Pre-publication history

The pre-publication history for this paper can be accessed here:

http://www.biomedcentral.com/1471-2474/15/47/prepub
